# Effect of Using Prickly Pear Seed Cake (*Opuntia ficus indica* L.) on Growth Performance, Digestibility, Physiological and Histometric Parameters in Rabbits

**DOI:** 10.3390/vetsci11100513

**Published:** 2024-10-17

**Authors:** Nadia Benali, Rafik Belabbas, Mounira Sais, Hacina AinBaziz, Baya Djellout, Fatima Nouara Ettouahria, Nadira Oulebsir, Gabriele Brecchia, Alda Quattrone, Giulio Curone, Laura Menchetti

**Affiliations:** 1 Laboratory of Reaserch “Health and Animal Productions”, Higher National Veterinary School, Road Issad Abes, Oued Smar, Algiers 16000, Algeria; n.benali@ensv.dz (N.B.); r.belabbas@ensv.dz (R.B.); h.ainbaziz@ensv.dz (H.A.); b.djellout@ensv.dz (B.D.); 2 Laboratory of Biotechnologies Related to Animal Reproduction, Institute of Veterinary Sciences, University Blida, B.P 270, Road of Soumaa, Blida 09000, Algeria; 3 Technical Institute of Animal Breeding, Bab Ali 16000, Algiers 16111, Algeria; s.alae@yahoo.fr; 4 Zéralda Educational Farm, Alger 16111, Algeria; fatimanouara42@gmail.com; 5 Nopaltec, Soug Ahras 41000, Algeria; n_oulebsir@yahoo.fr; 6 Department of Veterinary Medicine and Animal Sciences, University of Milan, Via dell’Università 6, 26900 Lodi, Italy; gabriele.brecchia@unimi.it (G.B.); giulio.curone@unimi.it (G.C.); 7 School of Biosciences and Veterinary Medicine, University of Camerino, Via Circonvallazione 93/95, 62024 Matelica, Italy

**Keywords:** blood metabolites, carcass yield, digestibility, growth, prickly pear seed cake, rabbits

## Abstract

Prickly pear (*Opuntia ficus indica* L.) could be used in rabbit nutrition in compliance with circular economy principles, global warming issues, and reduction of production costs. This study aims to evaluate the effects of dietary incorporation of prickly pear seed cake (PPSC) on growth, physiological, and histometric parameters in rabbits. A total of 105 rabbits were divided into three experimental groups (n = 35) and fed different diets: a commercial feed (C group), the same feed with alfalfa replaced by PPSC at 10% (10PP group), and at 20% (20PP group). They were group-housed in cages with 5 animals per cage from weaning until slaughtering. While body weights and weight gains were similar in all groups, the coefficients of nutrient digestibility of dry matter, fibers, and ashes, as well as the characteristics of intestinal villi, were improved in the 10PP group compared to the others (*p* < 0.05). The 20PP group showed a reduction in perirenal and interscapular fat (*p* < 0.05), as well as lower plasma concentrations of triglycerides and cholesterol compared to the C group (*p* < 0.001). In conclusion, PPSC can be incorporated into the diets of growing rabbits up to 20% as a partial substitute for alfalfa without the impairment of growth performance. Additionally, the inclusion of PPSC enhanced nutrient digestibility and increased the intestinal absorption surface area.

## Simple Summary

The prickly pear (*Opuntia ficus indica* L.) is a hardy plant well-adapted to hot and arid environments, rich not only in fiber but also soluble carbohydrates, lipids, minerals, and other antioxidant compounds. It has recently attracted attention for its potential uses in human and animal nutrition, however its effects on rabbits are still poorly understood. Its incorporation into the rabbit diet as a source of fiber would reduce the use of alfalfa with several benefits for the sustainability of rabbit farming from the point of view of the circular economy, global warming, and meat production costs. This study investigated the use of prickly pear seed cake in the rabbit’s diet with different inclusion percentages (10% and 20%). Various parameters were evaluated, including growth performance, nutrient digestibility, blood biochemical parameters, morphology of intestinal villi, and carcass characteristics. While productive parameters were similar, diets containing prickly pear seed cake led to improved digestive utilization of nutrients, as well as reduced blood lipid concentrations and fat content in the rabbit carcasses. The encouraging findings of our study suggest that the inclusion of prickly pear in rabbit feed is feasible, although further research is necessary to validate the current outcomes and to evaluate the economic convenience of its use.

## 1. Introduction

The rabbit is a herbivorous animal that requires a high fiber content in its diet to maintain a healthy gastrointestinal system and to effectively perform caecotrophy [1]. Dietary fibers are crucial components of the rabbits’ diet, essential for their health, welfare, and optimal growth. Both the quantity and quality of fiber are important for these aspects. Digestible fibers serve as substrate for microbial fermentation in the cecum, while indigestible fibers play a key role in promoting intestinal motility [2]. Moreover, adequate fiber intake supports proper feed conversion ratio by influencing intestinal motility and permeability, digestive and fermentative processes, nutrient absorption, intestinal microbiota development, and caecotrophy [2,3,4]. Consequently, an inappropriate diet lacking in sufficient quantity and quality of fibers can lead to various digestive disorders. This results in economic losses for breeders due to reduced growth rates, poor feed conversion ratio, and also increased mortality [5,6]. Therefore, it is essential to carefully formulate rabbit feed to include an adequate amount of fiber, ensuring the correct balance between digestible and indigestible fibers.

In Algeria, pelleted rabbit feed is often deficient in fiber [7]. To achieve the optimal fiber content in rabbit pellets, the addition of dehydrated alfalfa is typically required. However, alfalfa Algerian production is limited due to its high water requirements. Consequently, most alfalfa must be imported at high and continuously rising prices on the international market. This situation negatively impacts the Algerian rabbit sector by increasing feed costs, which, in turn, significantly raises the production cost of rabbit meat on the national market [8]. Feed expenses alone account for an estimated 60 to 70% of the total production cost [9]. A new strategy that incorporates the use of by-products to reduce water consumption and feed production costs, while maintaining adequate fiber levels, should be found. This approach could offer significant economic benefits and help mitigate the impact of climatic change, particularly in countries with hot and arid climates. In the last decades, several studies have examined the replacement of imported alfalfa with locally available raw materials, including raw olive cake [10,11], dried sulla [12], beans and peas [13], fig leaves [14], and brewer’s grains [15].

In addition, other local resources like the prickly pear (*Opuntia ficus indica* L.) and its by-products have recently attracted interest due to their beneficial effects on both humans and animals [16,17,18,19]. However, in Algeria, the use of prickly pear in livestock feed remains under-exploited compared to other countries such as Tunisia, Morocco, Egypt, and Brazil. The prickly pear is a hardy plant well-adapted to hot and arid environments [20], capable of rapid growth in arid and semi-arid regions. In Algeria, prickly pear plantations cover approximately 100,000 hectares [21]. Over the past decade, this plant has significantly grown in the steppes, generating employment opportunities and producing a wide range of products. These products include prickly pear seed oil, cake obtained from pressing the seeds, pulp, dried flowers, and cladode powder. In terms of chemical composition, the cactus cladode, the fruit, and the fruit pulp are rich in water, soluble carbohydrates, lipids, fiber, and minerals [22,23,24,25,26,27]. Overall, the nutritional and health benefits of the fruit are largely attributed to its antioxidant properties, resulting from the presence of ascorbic acid, polyphenols, flavonoids, and the mixture of yellow betaxanthin and red betacyanin [28].

Several studies have investigated the use of prickly pear as both a feed supplement and a substitute for maize, barley, and soybean meal in the diets of various animal species, including ruminants [17,29,30], poultry [19,31,32,33], and camelids [34]. These studies have employed different parts of the prickly pear, such as fruit pulp, bark, cladodes, and seed cake after oil extraction. The seed cake of prickly pear has been used primarily in the diets of broilers [19] and lambs during the fattening period [30]. Research on prickly pear use in rabbits is still limited, with most studies focusing on the digestibility of diets supplemented with its by-products [35,36,37]. Only few studies have investigated the effects of prickly pear on the rabbits’ physiological parameters, growing performance, and meat quality [38,39,40].

Therefore, the present study was conducted to evaluate the effects of different levels of inclusion of prickly pear seed cake as a partial substitute for alfalfa on the rabbit growth performance, nutrient digestibility, metabolic profile, carcass yield, and intestinal villi histometry.

## 2. Materials and Methods

### 2.1. Animals and Management

The trial was conducted at the rabbitry of the experimental station of the Technical Livestock Institute (ITELV) located in Baba-Ali in the commune of Birtouta (Algiers, Algeria), from May to July 2023.

**Figure 1:**
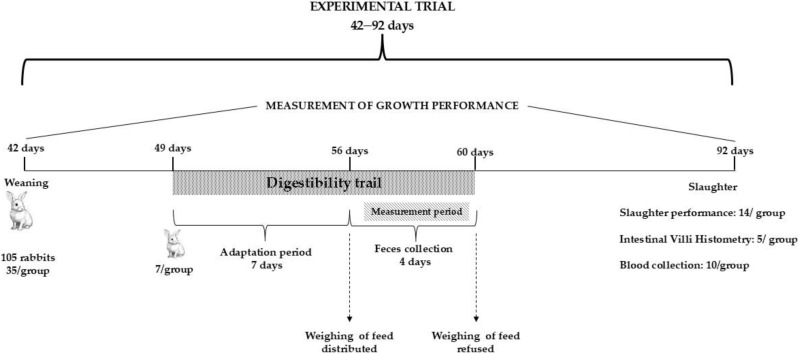
Experimental design.

This study was approved by the scientific council of the Health and Animal Productions laboratory, part of the Higher National Veterinary School (Algiers, Algeria). A total of 105 weaned kits of mixed sexes were used in the study. The characteristics of this genetic line (Synthetic line ITELV 2006) have been described by Ezzeroug et al. [41]. The rabbits were housed in collective (5 rabbits per cage) galvanized wire cages (59 cm × 54 cm × 35 cm) equipped with feeders and automatic nipple watering systems. After a one-week adaptation period, at 42 days of age, with an initial average weight of 1006 ± 17 g, the animals were divided into three groups, each with 7 replicates and 5 rabbits per cage (Figure 1). The rabbits were fed three different pelleted diets (Table 1 and Table 2) over a 7-week period, from weaning (42 days) until slaughtering (92 days): commercial diet (Control, C group), commercial diet supplemented with 10% (10PP group), and with 20% (20PP group) of prickly pear seed cake (PPSC) replacing an equivalent amount of alfalfa. Feed and water were provided ad libitum. The temperature and humidity were recorded 3 times a day, at 8 a.m., 12 p.m. and 3 p.m., and the lighting duration was 14 h per day. Throughout the experiment, the average temperature and humidity were 17.7 °C and 79%, respectively.

### 2.2. Growth Performance Evaluation

During the fattening period (from 42 to 92 days), live body weight (BW g) and feed intake (g/day; calculated as: distributed feed—refused feed/number of days) were recorded weekly. Average daily gain (ADG, g/day; calculated as: final BW–initial BW) and feed conversion ratio (FCR, g feed/g gain; calculated as: feed intake/average weight gain) were also determined.

### 2.3. Digestibility Trail

Nutrient digestibility was assessed at 49 days of age using the European reference method described by Perez et al. [42]. Twenty-one rabbits belonging to the three different experimental groups (C, 10PP, and 20PP; 7 rabbits/group) were individually housed in cages with a feces collection system. Following a 7-day adaptation period during which the animals were fed ad libitum, the nutrient digestibility trial was conducted over 4 consecutive days. During this period, feces were collected daily at 10 a.m., and were consequently weighed and stored at −20 °C for subsequent analysis. The feed was weighed on the first day of the trial at 10 a.m., and the amount of feed refused was measured on day 4 (end of the trial), at the same time. The apparent digestibility coefficient was calculated as follows: ADC%: ingested − excreted/ingested × 100.

### 2.4. Chemical Analyses

The chemical composition of PPSC, dehydrated alfalfa, experimental diets, and feces was analyzed according to AFNOR methods [43]. Dry matter (DM) was determined by desiccation in a circulating-air oven at 103 °C, ash (A) after incineration of organic matter in a muffle furnace at 550 °C, crude protein (CP) by Kjeldahl’s method, ether extract (EE) by a Soxhlet extractor, crude fiber (CF) by Weende method, and fiber fractions (NDF, ADF, ADL) were determined using Van Soest methods. The digestible energy of the feed was estimated using the Lebas prediction equation [44]. The apparent digestibility coefficients (ADC) of different ration components (dry matter, ash, crude protein, crude fiber, and ether extract) were calculated as follows:

ADC% DM = (Intake (DM) − Excretion (DM))/Intake (DM) × 100; ADC% Ash = (Intake (DM) × ash − Excretion (DM) × ash)/Intake (DM) × ash × 100; ADC% CP = (Intake (DM) × crude protein − Excretion (DM) × crude protein)/Intake (DM) × crude protein × 100; ADC% CF = (Intake (DM) × crude fiber − Excretion (DM) × crude fiber)/Intake (DM) × crude fiber × 100; ADC% EE = Intake (DM) × ether extract − Excretion (DM) × ether extract/Intake (DM) × ether extract × 100.

### 2.5. Slaughter Performances

At 92 days of age, fourteen rabbits of mixed sexes from each group (2 per cage representing the average weight of the group) were slaughtered by bleeding without prior fasting, to evaluate carcass characteristics following the guidelines of Blasco et al. [45]. Measurements of different segments of the digestive tract were conducted on 5 rabbits (1 per cage) of mixed sexes, representing the average weight of each group. Once the digestive tract segments had been emptied, they were washed and then wrung out by application to a clean cloth. Empty weights were determined for the stomach, small intestine, cecum, and colon.

### 2.6. Blood Collection and Analyses

Blood samples were collected from rabbits that had been fasted for 12 h. At slaughter, 92 days age, blood was drawn from 10 rabbits of mixed sexes (we selected 10 rabbits per group representing the average weight of the group), using heparin tubes. The blood samples were then centrifuged, and plasma was frozen at −20 °C for subsequent analysis of glucose, total proteins, triglycerides, cholesterol, urea, and creatinine, using Biolabo assay kits (Algeria).

### 2.7. Intestinal Villi Histometry

Five rabbits from each group (1 per cage, representing the average weight), aged 92 days were used for sampling. Samples were taken from the small intestine, specifically from the midpoint of each segment: duodenum, jejunum, and ileum, representing 1/5, 3/5, and 1/5 of the total length of the small intestine, respectively [46]. The intestinal samples were fixed in 4% buffered formalin for 48 h, and processed histologically following the methods described by Gabe [47]. Villi measurements were performed using an optical microscope (Motic type; magnification ×10) equipped with an image analysis software (Motic Image plus 2.0).

### 2.8. Preparation of Prickly Pear Seed Cake

The prickly pear seed cake (PPSC) used in our trial was sourced from a private processor located in Zéralda (Algiers, Algeria). The fruit seeds were obtained from the Bejaïa region and harvested in the summer of 2022. After cold oil extraction, the cake was stored in fiber bags, protected from light and moisture to preserve its nutritional content. The chemical composition of the PPSC is presented in Table 1. The cake was then transported to a livestock feed manufacturing unit located in Bouzaréah (Algiers) for the production of the experimental diets, which were formulated using Allix 3 software (A-Systems). The control diet (C) contained wheat bran, barley, corn, dehydrated alfalfa, and soybean meal. The other two diets were formulated to replace alfalfa with PPSC at incorporation rates of 10% (10PP) and 20% (20PP), while adhering to the nutritional recommendations for growing rabbits (De Blas and Mateos, 2010) [48]. The centesimal and chemical composition of the experimental diets is presented in Table 2.

### 2.9. Statistical Analysis

The parameters related to growth performances, carcass and digestive tract characteristics were analyzed using linear mixed models (LMMs). To evaluate BW, feed intake, and FCR, the models included the cage as a random effect and time as a repeated factor with an exchangeable correlation structure. These LMMs evaluated the main effects of time (7 levels for BW, 6 for feed intake and FCR), group (3 levels: C, 10PP, and 20PP), and the interaction between the group and time. Only group effect was included to evaluate total weight gain, carcass, and digestive tract characteristics. In the LMMs evaluating the characteristics of the intestinal tract (12 samples for each animal were evaluated), the rabbit was included as a random effect. Sidak adjustment was used for carrying out multiple comparisons. Results were expressed as means and standard error (SE).

Due to the smaller sample size and non-longitudinal nature of the observations, a more robust approach was used to analyze total feed intake, digestibility, morphometrics, and blood parameters. In particular, the distribution across groups was evaluated by Kruskal-Wallis tests, while multiple comparisons were adjusted with Bonferroni’s method. Results were expressed as the median, first (Q1), and third (Q3) quartile.

Statistical analyses were performed with SPSS Statistics version 25 (IBM, SPSS Inc., Chicago, IL, USA). Statistical significance was set at *p* < 0.05.

## 3. Results

### 3.1. Digestibility of the Diets

The digestibility of dry matter (DM), ether extract (EE), crude fiber (CF), and ashes (ash) of the diets were significantly different among groups (DM: *p* = 0.003, EE: *p* = 0.041, CF: *p* = 0.009, Ash: *p* = 0.026), as shown in Table 3. Specifically, rabbits belonging the 10PP group showed higher DM and CF digestibility coefficients compared to those in the 20PP group (10PP vs 20PP: +9.60% and 32.85%, *p* < 0.05), and higher ash digestibility than rabbits in group C (10PP vs. C: +5.62%; *p* < 0.05). However, the EE digestibility coefficient in the 10PP group was lower compared to rabbits in group C (10PP vs. C: −4.80%; *p* < 0.05). The digestibility coefficients of CP (Crude Protein) and DP (Digestible Protein) were not affected by the inclusion of PPSC at 10% and 20%.

### 3.2. Growth Performance

#### 3.2.1. Body Weight and Weight Gain

Regardless of group, the body weight increased significantly over time from 1006 ± 17 g on day 42 up to 2382 ± 17 g on day 92 (*p* < 0.001). The group effect was not significant, but interaction group × time indicated differences in the pattern of BW between groups (*p* = 0.044). In particular, the pairwise comparisons showed that at day 70, the 10PP group had a greater BW than C (+5.62%; *p* = 0.015; Table 4).

Weight gain (Table 5) decreased over time (*p* < 0.001), but no influence of the group (*p* = 0.135) or its interaction with time (*p* = 0.593) was found. Even multiple comparisons did not find differences in individual time points. However, the total weight gain between post-weaning and slaughter (42–91 days) was higher in the 10PP group than in the control group (1.33 ± 0.03 kg, 1.39 ± 0.03 kg, and 1.43 ± 0.03 kg for groups C, 20PP and 10PP, respectively; *p* = 0.049).

#### 3.2.2. Feed Intake and FCR

The recorded results revealed that feed intake (Table 6) and FCR (Table 7) were influenced by time (*p* < 0.001) and their interaction with the group (*p* = 0.022 and *p* = 0.027 for feed intake and FCR, respectively). Feed intake increased progressively over time, and a significant difference (*p* = 0.049) was noted during the period of 84–91 days of age, when animals in group C had a significantly higher feed intake compared to the rabbits in 10PPgroup (C vs. 10PP: +11.89%; *p* < 0.05). Also, during the same period, animals in group C recorded a higher FCR (a difference of +21.47%; *p* < 0.001). However, total food consumption (Mdn = 5886, Q1 = 5630, Q3 = 5998 g for group C; Mdn = 5830, Q1 = 5483, Q3 = 6071 g for 10PPgroup; Mdn = 5951, Q1 = 5590, Q3 = 6052 g for 20PPgroup; *p* = 0.754) and FCR (Mdn = 3.8, Q1 = 3.0, Q3 = 4.8 g for group C; Mdn = 3.9, Q1 = 3.0, Q3 = 4.8 g for 10PPgroup; Mdn = 3.5, Q1 = 2.9, Q3 = 4.7 g for 20PP group; *p* = 0.749) did not differ between groups.

### 3.3. Carcass Characteristics

The proportions of WPF/BW (weight perirenal fat/body weight) and WIF/BW (weight interscapular fat/body weight), as well as WIF (weight interscapular fat), were lower in rabbits from the 20PP group compared to group C (20PP vs. C: −19.64%, −21.42%, and −16.66%, respectively; *p* < 0.05), while rabbits in 10PPgroup recorded intermediate values without significant effect (Table 8).

### 3.4. Digestive Tract

#### 3.4.1. Weight of Digestive Tract

The results of the weight of the digestive tract are shown in Table 9. No significant effect of the incorporation of the PPSC was revealed except for the WES/BW (weight stomach/body weight) ratio (*p* < 0.05), although multiple comparisons did not reveal any differences.

#### 3.4.2. Histometry of Intestinal Villi

The results of the histometry of the intestinal villi are reported in Table 10. The rabbits in group 10PP recorded higher values than those in groups C and 20PP for all measured parameters (*p* < 0.05), except for the base of the duodenum, which was similar to that of the 20PP group.

### 3.5. Blood Parameters

The rabbits in the 20PP group recorded significantly lower triglyceride and cholesterol levels compared to those in groups C and 10PP (*p* < 0.05) Specifically, triglyceride levels were reduced by −34.86% relative to the C and 10PP groups, while cholesterol levels decreased by −20.31% compared to the C group (Table 11).

## 4. Discussion

### 4.1. Composition of Prickly Pear Seed Cake

The prickly pear seed cake (PPSC) can be considered a valuable resource for rabbit diets, offering a higher fiber content compared to dehydrated alfalfa. However, the CP and DE content of alfalfa is higher than those of PPSC (18.38% and 2145 kcal/kg vs. 7.77% and 1159 kcal/kg). The low energy concentration of PPSC is probably linked to its high fiber concentration. Indeed, studies have shown that high fiber content can reduce the energy concentration of the feed [2,49], although this effect may vary depending on the quality of the product. Masmoudi et al. [50] reported that seed cakes from prickly pears harvested between August and November had lower levels of CP, NDF, ADF, and ADL, compared to those used in our study (−19.53%, −21.25%, −28.96%, and −11.53%). On the other hand, the DM content was higher (+5.97%) compared with the DM of the PPSC in our experiment. The variation in the chemical composition of PPSC can be attributed to several factors: the plant’s origins (including the climate in which it is grown), agronomic practices (tillage, fertilization, and irrigation) [51], genetic differences [52,53], and likely the manufacturing process and the extent of oil extraction.

### 4.2. Effect on Live Weight and Weight Gain

Based on the results of this study, the incorporation of 10% PPSC in the rabbit feed resulted in an improved average daily gain. This improvement might be linked to the nutrients present in prickly pear seeds, particularly the proteins, which have a composition similar to that of sunflower [54,55]. Prickly pear seeds are also rich in essential amino acids, including methionine, cysteine, lysine, tryptophan, and arginine, which are particularly important for the rabbit’s growth and development [56]. In addition, these seeds contain important minerals such as Ca, P, Na, Cl, Mg, Fe, Cu, and Zn [54,57,58]. Moreover, prickly pear seeds exhibit antioxidant, anti-inflammatory, and anti-microbial properties, thanks to their content of flavonoids, polyphenols, and tannins [54,55,59]. Furthermore, the high levels of polyunsaturated fatty acids, particularly linoleic acid (C18:2, ω6) and oleic acid (C18:1, ω9) [60], may help modulate the immune response.

Our results are consistent with those reported by Cherif et al. [19], who observed increased broiler weight gain with a 10% incorporation of PPSC in the diet. Conversely, Djeballah et al. [30] reported a decrease in lamb weight with higher PPSC incorporation levels (15, 30, and 45%). Compared to other cactus products, studies have shown that incorporating cladodes and fruit peels into the diet results in greater weight gain in both rabbits [36,38,40] and broilers [19,61,62]. Specifically, rabbits fed a diet with 75% prickly pear peels replacing hay showed the highest values of live body weight and total weight gain compared to those in the control, 25%, and 50% groups [36]. Zeedan et al. (2015) also reported a significant increase in body weight and total weight gain in rabbits fed diets containing 30% prickly pear cladodes, in comparison to the other experimental groups [38]. Another study on fattening rabbits aged from 5 to 13 weeks found that the dietary replacement of barely with 50% prickly pear fruits and 50% prickly pear peels lead to a noticeable improvement in body weight gain, although it did not affect live body weight, compared to the other experimental groups (control and 25% of integration) [39]. Finally, it was reported that dietary supplementation with 30% prickly pear peel resulted in a greater increase in both body weight and body weight gain in fattening rabbits compared to the inclusion levels of 10 and 20% as well as the control groups [40].

### 4.3. Effect on Feed Intake and Feed Conversion Ratio

In our study, the inclusion of PPSC did not affect feed intake or feed conversion ratio (FCR). The composition of the PPSC, in particular its high fiber content, might have influenced the rabbits’ feed intake, as dietary fiber is known to regulate food consumption in this species [63,64]. According to Gidenne [65], the rabbit’s voluntary food consumption is influenced by the dietary ADF content, due to the low digestibility of this fiber fraction. Additionally, the ADF content acts as a ‘ballast’ that likely limits food intake. Gidenne and Lebas [66] reported that when the dietary fiber content is very high (ADF), the animal cannot sufficiently increase its feed intake. Furthermore, Gidenne [67] noted that when rabbits are fed fiber-rich diets, and their nutritional requirements are fully met, their intake levels tend to remain relatively stable. Our results differ from those observed in broiler chickens and lambs [19,30], whose feed intake and FCR decrease with increasing PPSC inclusion (10%, 20% and 30%, 45%). El-Neney et al. [40] and Bakr [36] reported a decrease in daily feed intake as well as an increase in FCR in rabbits fed increasing levels of cladodes and fig bark. The supplementation of the rabbits’ diet with 30% prickly pear cladodes significantly reduced the feed intake and FCR [38]. An improvement in the FCR, but not in the feed intake was observed in another study, in which the diet of fattening rabbits was integrated with 50% prickly pear fruits or 50% prickly pear peel, in comparison to other experimental groups (25% of prickly pear fruit or peel and control) [39].

The same was observed in broiler chickens fed diets containing prickly pear seeds [62]. On the other hand, in Japanese quail, Regab [31] observed an improvement in feed intake with the dietary incorporation of prickly pear bark.

### 4.4. Effect on Digestibility

Our results showed that the digestibility of DM and CF decreased when PPSC was incorporated into the diet at 20%. This decrease is likely related to the high indigestible fiber content in the diet with 20% PPSC (22.08% ADF). According to Gidenne [68], Gidenne et al. [69] and De Blas et al. [70], the increase in fiber reduces the residence time of feed, which is relatively short in the cecum of rabbits [71]. Studies involving the incorporation of olive pulp and olive cake into the basal diet at levels of 20% have shown a reduction in the digestibility of organic matter, crude protein, and fiber [11,72]. Ash digestibility was higher in the 10% PPSC diet, probably due to the mineral content of the seed cake [54]. Our results are in agreement with findings other studies that evaluated the effects of diets with varying percentages of different prickly pear by-products [36,38,39,40]. Backr (2019) reported improved digestibility coefficients and nutritive values (DM, CP, CF, TDN, and DE) in rabbits fed diets integrated with prickly pear peels at different levels (25%, 50%, and 75% or 10%, 20%, and 30%, respectively) and prickly pear cladodes, compared to those fed the control diet [36,38,40]. On the contrary, another study found that the digestibility was not affected by the diet of the fattening rabbits that were fed different percentages of prickly pear fruits and peels compared to the control diet [39].

### 4.5. Effect on Slaughter Yield and Carcass Characteristics

Incorporating prickly pear seed cake (PPSC) at levels of 10% and 20% did not affect the rabbits’ carcass characteristics or yield. However, the carcass adiposity significantly decreased in rabbits fed diets containing 20% PPSC. This result could be associated to the high content of insoluble fiber (ADF) in PPSC. Fibre is known to stimulate the intestinal activity in rabbits, which can increase calories expenditure and reduce fat accumulation [73]. The insoluble dietary fiber present in PPSC may also enhance satiety in rabbits, potentially reducing their appetite and leading to lower feed consumption [74,75]. This could contribute to a reduction in the adiposity. In fact, we found that rabbits in our trial did not increase their feed intake compared to the control group when the PPSC level was increased to 20%. In addition, the adiposity reduction may be linked to the lower digestible energy of the diet containing 20% PPSC compared with to control diet (11.40 MJ/DM vs. 11.97 MJ/DM). Insoluble fiber is not fully digestible, so some of the energy contained in fibre-rich feed may be lost in the feces rather than being absorbed. Farías-Kovac et al. [49] found that increasing dietary fibers led to a progressive reduction in the energy retained in tissues. The low adiposity recorded in rabbits fed the 20% PPSC diet can also be attributed to the low concentration of triglycerides and cholesterol, both considered as indicators of energy reserves [76].

Our results are partially consistent with those of other studies, which have demonstrated that using various cactus by-products can lead to reduced adiposity and improved carcass yield [36,38,39]. Amer et al. (2019) reported a reduction in abdominal fat in rabbits fed a diet containing 50% prickly pear peels compared to the control. However, they also observed an increase in the weight of liver, heart and edible giblets [39]. The inclusion of prickly pear peels in the diet of fattening rabbits at the percentage of 25%, 50%, and 75% or prickly pear cladodes (10%, 20%, and 30%) increased the dressing percentage and carcass weight [36,38]. In contrast, Pascoal (2020) reported no significant effects of including forage cactus meal at various percentages on hot and cold carcass weight, carcass yield, viscera weight, and liver weight [37]. Studies on other animal species, including kids [77,78], lambs [79], and broilers [32] reported decreased adiposity when animals consumed cactus-based diets.

### 4.6. Effect on Digestive Tract Weight

In our study, the incorporation of PPSC had no effects on the weight or proportions of the various segments of the intestinal tract in relation to the live weight. The only exception was found regarding the WES/BW proportion, which decreased in rabbits fed with 10% and 20% PPSC, despite the high fiber content of these diets (20.22% and 22.08% ADF). The lower stomach weight observed in the 10PP group may be related to the reduced feed intake observed in rabbits between 84 and 91 days. According to Anguita et al. [80], the fiber in the diet generally increases the weight of the digestive tract, with the most pronounced effects in the stomach and colon, while the small intestine is less affected [81,82].

Given the limited research on the effects of prickly pear and its by-products on the rabbit digestive tract, we investigated other fiber-rich alternatives. Different studies investigated the effects of wheat screening residue [83] and beet pulps [84] on the digestive tract in rabbits, observing an increase in stomach and caecum weight. On the other hand, Chao and Li [85], Wu et al. [86], and Nan Bin et al. [87] investigated the impact of different fiber levels in the diet, reporting higher stomach, caecum, and intestine weights with 25% ADF, 25.39% CF, and 22.37% CF, respectively. In our study, the absence of an effect of PPSC on the digestive tract could be related to the specific botanical origin of the fiber, as noted by Philippe et al. [88], as well as to the level and type of dietary fiber that plays a crucial role in the development of the digestive tract [89].

### 4.7. Effect on Blood Biochemical Parameters

The diet containing 20% PPSC significantly reduced plasma triglyceride and cholesterol levels compared to the control group. The reduction in these lipid parameters may be attributed to specific compounds present in PPSC that can influence lipid metabolism in rabbits. Indeed, Louacini et al. [90] demonstrated that the pectin found in Opuntia can lower cholesterol levels by binding to bile acids, thereby promoting cholesterol catabolism. Wolfram et al. [91] also attributed the hypolipidemic effects of cactus to the pectin in its pulp, which reduces lipid absorption and increases fecal sterol excretion. Another factor contributing to cholesterol reduction may be the fiber content. As previously mentioned, dietary fiber can induce satiety, potentially reducing overall cholesterol intake due to decreased food consumption. In addition, the interaction between flavonoids, betaines, and vitamin E appears to be at the root of the prickly pear’s hypolipidemic activity [92]. Our results are in agreement with several studies conducted in different animal species, including chickens fed prickly pear peel and cladodes [32,61], as well as research on ewes fed cladodes with and without thorns [90]. Our results also corroborate those recorded in rabbits [36,39] fed prickly pear fruits and peel. Specifically, fattening rabbits fed prickly pear peels at different percentages (25%, 50%, and 75%) showed reduced plasma concentrations of ALT, AST, total lipids, total cholesterol, HDL, and LDL, as well as increased levels of total proteins, albumin, and globulin [36]. In another study on rabbits, dietary supplementation with prickly pear peels and prickly pear fruits resulted in decreased plasma triglycerides, cholesterol, and LDL levels, while HDL concentrations were increased compared to the control group [39]. Finally, El-Neney et al. (2019) reported that increasing the percentage of prickly pear peels in the diet of fattening rabbits led to higher plasma concentrations of total protein, albumin, and globulin, while total lipid levels were reduced compared to the control group.

### 4.8. Effect on the Histometry of Intestinal Villi

The incorporation of PPSC at 10% increased the height, base, and surface area of the villi in the small intestine. It should be noted that the increase in villi height, base, and surface area observed with the 10PPSC diet may be related to the natural growth-stimulating properties of the dietary fibers. These fibers play a crucial role in regulating the intestinal flora, maintaining the intestinal mucosa, and preserving the integrity of the intestinal mucus [70]. Specifically, the ADF fiber has been shown to stimulate the growth of intestinal villi by promoting cell proliferation [93] and improving the overall health of the intestinal tract [65,66,68,69]. Satchithandam et al. (1990) [94] and Piel et al. [95] observed that fiber-rich diets increase the number of goblet cells, enhance mucin secretion, and improve luminal content density, all of which contribute to increased nutrient absorption in rats and pigs.

On the other hand, high fiber content can adversely affect villi development, as reported by Alvarez et al. [96] with 36% ADF, Chao and Li [85] with 25% ADF, and Zanetti et al. [97] with 43.71% ADF. In our study, it appears that the reduced height, base, and surface area of the intestinal villi in rabbits fed the 20PPSC diet might be attributed to the high ADF content (22.08%), which is considered excessive and likely adversely affected villi growth.

## 5. Conclusions

This study highlights the potential of incorporating the prickly pear seed cake up to 20% as a source of fiber in the rabbit diet. Its incorporation enhanced weight gain in fattening rabbits and increased the digestive utilization of nutrients by improving the intestinal villi’s absorption surface area. Additionally, it lowered blood lipid concentrations and reduced carcass fat, although its impact on some other productive parameters was limited. These preliminary results suggest that prickly pear seed cake could be a valuable addition to rabbit nutrition, potentially offering a new strategy to reduce alfalfa imports in Algeria, thereby lowering the rabbit’s feed costs, improving the profitability of breeders, and enhancing animal welfare. However, further studies with a larger number of animals are required to: (i) evaluate the mechanism affecting growth performance; (ii) verify the impact on the rabbit meat quality; and (iii) conduct a cost-analysis to assess the sustainability of its use in rabbit breeding.

## Figures and Tables

**Table 1:** Chemical composition of prickly pear seed cake (PPSC) and dehydrated Alfalfa (on dry matter basis).

Items (%); kcal/kg et MJ/kg	DM	Ash	CP	EE	CF	NDF	ADF	ADL	DE *
PPSC	91.86	1.50	7.77	3.68	69.62	92.13	64.25	39.62	1159/4.89
Alfalfa dehydrated	91.57	9.78	18.38	1.97	31.63	68.14	34.91	15.28	2145/8.97

PPSC: prickly pear seed cake; Dry matter (DM), ashes (Ash), crude protein (CP), crude fiber (CF), ether extract (EE), neutral detergent fiber (NDF), acid detergent fiber (ADF), acid detergent lignin (ADL), Digestible energy (DE) *: was calculated according to Lebas (2013) using the following equation: * DE = 15.627 + 0.000982 CP² + 0.0040 EE² − 0.0114 MM² − 0.169 ADF ± 1.250 MJ/kg DM. * DE in M Joules /kg; DM = Dry matter; CP = % crude protein in DM; EE = % ether extract (lipids) in DM; MM = % minerals (ash) in DM; ADF = % acid detergent fiber in DM; CF = % crude fiber in DM.

**Table 2:** Ingredients and chemical composition of experimental diets.

	C	10PP	20PP
***Ingredients (%)***			
Maize	34.70	42.70	41.38
Wheat bran	20.00	4.00	7.00
Soybean meal	14.00	19.20	22.90
Alfalfa dehydrated	29.00	21.00	3.00
Prickly pear seed cake (PPSC)	0.00	10.45	22.10
Limestone	0.70	0.70	1.67
Dicalcium phosphate	0.45	0.80	0.80
Salt	0.15	0.15	0.15
Premix *	1.00	1.00	1.00
Total	100	100	100
***Chemical composition % (DM)***			
DM	88.72	89.40	89.52
CP	17.98	16.85	16.78
EE	2.99	3.52	3.71
Ash	8.55	8.35	8.43
CF	13.53	14.75	16.50
NDF	32.82	34.45	34.94
ADF	18.72	20.22	22.08
ADL	5.29	5.58	6.15
DE ^1^ (kcal/kg)	2861	2804	2725
DE (MJ/kg)	11.97	11.73	11.40

DM = Dry matter, CP = crude protein, EE = ether extract, Ash = ashes, CF = crude fiber, NDF = Neutral detergent fiber, ADF = Acid detergent fiber, ADL = Acid detergent lignin, ^1^ DE = Digestible energy, calculated according to Lebas (2013) using the following equation: DE = 15.627 + 0.000982 CP² + 0.0040 EE^2^ − 0.0114 MM² − 0.169 ADF ± 1.250 MJ/kg DM. DE in M Joules /kg DM; DM = Dry matter; CP = % crude protein in DM; EE = % ether extract in DM; MM = % ash in DM; ADF = % acid detergent fiber in DM; CF = % crude fiber in DM. * Premix: Vitamin A: 1,000,000 IU, Vitamin D3: 250,000 IU, Vitamin E: 2500 IU, Vitamin K3: 300 mg, Vitamin B1: 170 mg, Vitamin B2: 500 mg, Vitamin B3: 3000 mg, Vitamin B5: 1000 mg, Vitamin B6: 400 mg, Vitamin B9: 75 mg, Vitamin B12: 2000 mg, Biotin: 5000 mg, Choline: 57,000 mg, Iron: 6000 mg, Zinc: 6500 mg, Copper: 1500 mg, Manganese: 7000 mg, Iodine: 200 mg, Selenium: 40 mg, Sodium: 135 g, Chlorine: 201 g Methionine: 131,000 mg, Lysine: 19,000 mg.

**Table 3:** Parameters related to digestibility recorded in rabbits fed a control diet (C), supplemented with 10% (10PP) and 20% (20PP) prickly pear seed cake. Values are medians, first (Q1) and third (Q3) quartiles (n = 7).

	Groups									
	C			10PP			20PP			*p* Value
Parameters	Median	Q1	Q3	Median	Q1	Q3	Median	Q1	Q3	
DM, %	72.01 ^ab^	71.53	72.60	73.81 ^b^	72.41	75.49	66.72 ^a^	64.35	69.54	**0.003**
EE, %	81.83 ^b^	81.17	83.02	78.25 ^a^	76.75	79.77	80.35 ^ab^	79.53	80.71	**0.041**
CP, %	81.02	80.01	82.54	82.05	79.66	84.45	78.07	75.43	80.86	0.091
CF, %	26.77 ^ab^	22.16	29.17	33.69 ^b^	28.11	36.38	22.62 ^a^	21.90	26.15	**0.009**
Ash, %	73.44 ^a^	72.11	73.95	77.82 ^b^	77.32	80.08	75.38 ^ab^	69.18	78.90	**0.026**
DP * (g/100 g)	13.00	12.61	13.86	13.14	12.76	13.62	12.32	11.86	12.75	0.129

DM = dry matter; EE = ether extract; CP = crude protein; CF = crude fiber; DP = Digestible protein. DP * = CP * ADC (apparent digestibility coefficient) of CP. Values followed by the same letter in each row do not differ significantly (*p* < 0.05). Bold *p*-values denote statistical significance at the *p* < 0.05 level.

**Table 4:** Changes in body weight in rabbits fed a control diet (C), supplemented with 10% (10PP) and 20% (20PP) prickly pear seed cakes (n = 35). Values are means and standard errors (SE).

Day	Group					
	C		10PP		20PP	
	Mean	SE	Mean	SE	Mean	SE
42	1013	21	1006	22	998	20
49	1227	23	1251	26	1240	24
56	1449	25	1485	34	1455	29
63	1663	30	1738	30	1694	28
70	1834 ^a^	40	1966 ^b^	33	1876 ^a^	30
77	2031	33	2128	32	2040	37
84	2203	33	2284	35	2223	33
91	2344	35	2444	32	2377	35

Values followed by the same letter in each row do not differ significantly (*p* < 0.05).

**Table 5:** Weight gain of rabbits fed a control diet (C), supplemented with 10% (10PP) and 20% (20PP) prickly pear seed cakes (n = 35). Values are means and standard errors (SE).

Time	Group					
	C		10PP		20PP	
	Mean	SE	Mean	SE	Mean	SE
42–48 days	29.78	1.53	35.18	1.18	34.54	2.74
49–55 days	31.74	1.51	32.73	2.66	30.71	2.39
56–62 days	30.63	1.47	32.91	2.00	33.77	1.91
63–69 days	24.33	3.65	31.00	1.97	27.50	2.32
70–76 days	24.59	1.28	23.12	1.24	23.14	2.32
77–83 days	24.56	1.57	22.22	1.48	26.22	1.89
84–91 days	20.23	1.80	22.83	1.71	22.01	2.28

**Table 6:** Mean feed intake (g/d) of rabbits fed a control diet (C), supplemented with 10% (10PP) and 20% (20PP) prickly pear seed cake. Values are means and standard errors (SE).

Time	Group					
	C		10PP		20PP	
	Mean	SE	Mean	SE	Mean	SE
42–48 days	81	2	90	2	92	4
49–55 days	93	2	97	1	92	2
56–62 days	103	3	107	5	104	4
63–69 days	105	4	110	5	98	7
70–76 days	103	2	106	2	105	7
77–83 days	115	2	114	3	117	5
84–91 days	125 ^a^	8	110 ^b^	8	121 ^ab^	9

Values followed by the same letter in each row do not differ significantly (*p* < 0.05).

**Table 7:** Feed conversion ratio (g feed/g gain) of rabbits fed a control diet (C), supplemented with 10% (10PP) and 20% (20 PP) prickly pear seed cake. Values are means and standard errors (SE). Values followed by the same letter in each row do not differ significantly (*p* < 0.05).

Time	Group					
	C		10PP		20PP	
	Mean	SE	Mean	SE	Mean	SE
42–48 days	2.7	0.1	2.6	0.0	2.7	0.2
49–55 days	2.9	0.1	3.1	0.2	3.2	0.3
56–62 days	3.4	0.2	3.3	0.2	3.1	0.1
63–69 days	3.8	0.1	3.6	0.2	3.6	0.3
70–76 days	4.4	0.3	4.8	0.4	4.9	0.4
77–83 days	4.7	0.1	5.3	0.3	4.6	0.4
84–91 days	6.6 ^a^	0.7	5.0 ^b^	0.4	4.9 ^b^	0.5

**Table 8:** Parameters related to the characteristics of the carcass recorded in animals fed a control diet (C), supplemented with 10% (10PP) and 20% (20PP) prickly pear seed cakes. Values are means and standard deviations (SD) (n = 15).

	Group						
	C		10PP		20PP		*p* Value
Parameters	Mean	SE	Mean	SE	Mean	SE	
BW (g)	2412.71	47.94	2460.92	19.50	2455.50	31.29	0.553
SW (g)	241.40	6.73	255.41	8.40	253.10	6.22	0.328
SW/BW%	10.13	0.32	10.54	0.22	10.30	0.18	0.465
WHC (g)	1661.32	43.16	1780.13	25.25	1718.68	52.44	0.138
HC/BW	71.54	0.59	72.38	0.59	71.65	0.67	0.553
WL (g)	63.40	2.39	61.85	2.09	61.64	2.25	0.822
WL/BW%	3.80	0.16	3.73	0.12	3.67	0.15	0.813
WCC (g)	1626.46	27.17	1665.53	41.14	1688.46	30.41	0.424
WCC/BW%	69.48	0.87	69.26	0.52	68.21	0.42	0.322
WPF (g)	32.34	1.64	31.24	1.58	27.59	1.28	0.064
WPF/BW	1.34 ^b^	0.06	1.28 ^ab^	0.05	1.12 ^a^	0.04	**0.013**
WIF	8.05 ^b^	0.35	7.79 ^ab^	0.34	6.90 ^a^	0.33	**0.043**
WIF/BW	0.34 ^b^	0.02	0.32 ^ab^	0.01	0.28 ^a^	0.01	**0.011**
WDT	343.21	15.91	338.19	11.75	357.58	10.27	0.523
WDT/BW%	14.25	0.61	13.99	0.42	14.59	0.44	0.674

BW = body weight; SW = skin weight; WHC = weight hot carcass; WL = weight liver; WCC = weight cold carcass: the weight of the carcass after 24 h in a cool place at 4 °C; WPF = weight perirenal fat; WIF = weight interscapular fat; WDT = weight digestive tract. Values followed by the same letter in each row do not differ significantly (*p* < 0.05). Bold *p*-values denote statistical significance at the *p* < 0.05 level.

**Table 9:** Weight of the digestive tract recorded in animals fed a control diet (C), supplemented with 10% (10PP) and 20% (20PP) prickly pear seed cakes. Values are medians (Md), first (Q1) and third (Q3) quartiles (n = 5).

	Groups									
	C			10PP			20PP			
Parameters	Md	Q1	Q3	Md	Q1	Q3	Md	Q1	Q3	*p* Value
BW(g)	2317	2257	2352	2455	2449	2455	2475	2465	2498	0.102
WES(g)	19.70	19.60	22.20	16.40	15.90	18.80	18.00	16.70	18.80	0.087
WES/BW	0.87	0.84	0.96	0.69	0.65	0.73	0.72	0.67	0.74	**0.037**
WESI(g)	50.15	48.20	54.50	50.00	47.00	53.20	51.40	50.40	52.10	0.763
WESI/BW	2.16	2.09	2.30	2.09	1.92	2.09	2.04	1.91	2.08	0.196
WEC(g)	32.70	31.20	32.80	31.30	30.80	35.10	34.60	34.30	42.90	0.230
WEC/BW	1.42	1.38	1.53	1.25	1.22	1.43	1.40	1.26	1.75	0.379
WECol(g)	28.30	26.30	32.60	27.60	27.00	28.10	33.20	30.80	35.50	0.249
WECol/BW	1.22	1.01	1.39	1.13	1.10	1.18	1.30	1.26	1.35	0.330

BW = Body weight; WES = weight empty stomach; WESI = weight empty small intestine; WEC = weight empty ceacum; WECol = weight empty colon. Bold *p*-values denote statistical significance at the *p* < 0.05 level.

**Table 10:** Histometry of intestinal villi recorded in animals fed a control diet (C), supplemented with 10% (10PP) and 20% (20PP) prickly pear seed cakes. Values are means and standard errors (SE) (n = 5).

	Parameter	Group						
		C		10PP		20PP		*p*-Value
Segments		Mean	SE	Mean	SE	Mean	SE	
Duodenum	High (µm)	3132.79 ^b^	73.55	4004.77 ^a^	65.09	3631.79 ^b^	60.22	**<0.001**
	Base (µm)	502.37 ^b^	12.64	580.77 ^a^	11.60	566.10 ^ab^	12.24	**0.006**
	Area (mm^2^)	4961.99 ^b^	177.96	7255.58 ^a^	158.99	6428.76 ^b^	152.67	**<0.001**
Jejunum	High (µm)	3460.74 ^b^	67.86	4210.97 ^a^	90.47	3777.86 ^b^	61.64	**<0.001**
	Base (µm)	412.45 ^b^	9.91	558.72 ^a^	11.79	541.76 ^a^	8.09	**<0.001**
	Area (mm^2^)	4522.49 ^b^	157.58	7313.47 ^a^	176.74	6418.08 ^b^	137.18	**<0.001**
Ileum	High (µm)	2077.95 ^b^	43.72	3087.31 ^a^	50.01	2344.15 ^b^	47.87	**<0.001**
	Base (µm)	444.45 ^b^	8.46	510.76 ^a^	9.40	489.94 ^b^	9.34	**0.013**
	Area (mm^2^)	2901.31 ^b^	83.57	4910.05 ^a^	86.59	3609.52 ^b^	103.45	**<0.001**

Values followed by the same letter in each row do not differ significantly (*p* < 0.05). Bold *p*-values denote statistical significance at the *p* < 0.05 level.

**Table 11:** Blood parameters recorded in animals fed a control diet (C), supplemented with 10% (10PP) and 20% (20PP) prickly pear seed cakes. Values are medians, first (Q1) and third (Q3) quartiles (n = 10).

	Groups									
	C			10PP			20PP			*p* Value
Parameters	Median	Q1	Q3	Median	Q1	Q3	Median	Q1	Q3	
Glucose (mmol/L)	14.33	12.93	16.56	14.14	10.95	15.35	14.51	14.26	15.37	0.786
Triglycerides (mmol/L)	1.49 ^b^	1.44	1.51	1.45 ^b^	1.41	1.49	1.09 ^a^	1.04	1.12	**<0.001**
Cholesterol (mmol/L)	2.31 ^b^	2.09	2.53	2.06 ^ab^	1.94	2.10	1.92 ^a^	1.78	2.06	**0.002**
Total proteins (g/L)	60.00	58.80	64.60	63.05	57.20	68.30	55.75	54.20	59.90	0.098
Urea (mmol/L)	12.20	12.04	12.37	12.24	11.95	12.85	12.62	12.40	12.78	0.308
Creatinine (mg/L)	20.00	18.33	21.67	21.67	20.00	21.67	21.67	20.00	23.33	0.187

Values followed by the same letter in each row do not differ significantly (*p* < 0.05). Bold *p*-values denote statistical significance at the *p* < 0.05 level.

## Data Availability

The data generated and analyzed during this study are included in this article.
